# Thermomechanical stress analyses of nanowarming-assisted recovery from cryopreservation by vitrification in human heart and rat heart models

**DOI:** 10.1371/journal.pone.0290063

**Published:** 2023-08-16

**Authors:** Purva Joshi, Yoed Rabin

**Affiliations:** 1 Department of Mechanical Engineering, Biothermal Technology Laboratory, Carnegie Mellon University, Pittsburgh, PA, United States of America; 2 Department of Mechanical Engineering, Forbes Avenue, Pittsburgh, PA, United States of America; Cardiff Metropolitan University, UNITED KINGDOM

## Abstract

This study investigates thermomechanical stress in cryopreservation by vitrification of the heart, while exploring the effects of nanowarming-assisted recovery from cryogenic storage. This study expands upon a recently published study, combining experimental investigation and thermal analysis of cryopreservation on a rat heart model. Specifically, this study focuses on scenarios with variable concentrations of silica-coated iron-oxide nanoparticles (sIONPs), while accounting for loading limitations associated with the heart physiology, as well as the properties of cryoprotective agent (CPA) solution and the geometry of the container. Results of this study suggest that variable sIONP concentration based on the heart physiology will elevate mechanical stresses when compared with the mathematically simplified, uniform distribution case. The most dangerous part of rewarming is below glass transition and at the onset of nanowarming past the glass transition temperature on the way for organ recovery from cryogenic storage. Throughout rewarming, regions that rewarm faster, such as the chambers of the heart (higher sIONP concentration), undergo compressive stresses, while the slower rewarming regions, such as the heart myocardium (low sIONP concentration), undergo tension. Being a brittle material, the vitrified organ is expected to fail under tension in lower stresses than in compression. Unfortunately, the location and magnitude of the maximum stress in the investigated cases varied, while general rules were not identified. This investigation demonstrates the need to tailor the thermal protocol of heart cryopreservation on a case-by-case basis, since the location, orientation, magnitude, and instant at which the maximum mechanical stress is found cannot be predicted *a priori*. While thermomechanical stress poses a significant risk to organ integrity, careful design of the thermal protocol can be instrumental in reducing the likelihood of structural damage, while taking full advantage of the benefits of nanowarming.

## Introduction

Organ transplantation is one of the most impactful medical achievements, which has added over two million life-years to patients in the United States alone in the past quarter of a century [1]. Despite notable advances in medicine and biotechnology, a persistent gap remains between the supply and demand of organs for transplantation and medical research. By some accounts, if only one-half of the currently discarded hearts and lungs were utilized for transplantation, the waitlists for these organs could be eliminated within 2–3 years [2].

Preservation in very low temperatures (cryopreservation) is a promising technique that can potentially increase the availability of organs by improving both the logistics and outcomes of transplantation [3–13]. With this application, cryoprotective agents (CPAs) are loaded onto the biospecimen to control ice formation and growth. However, it becomes increasingly challenging to control and ideally circumvent ice formation using low concentration CPAs, where the complexity of the cryopreservation protocol increases with the specimen size [14–19]. The only viable approach to long-term cryopreservation of large organs, such as the heart, kidney, or liver appears to be by vitrification (*vitreous* in Latin means *glassy*), where ice formation is completely avoided, and the material is preserved in a solid-like amorphous state. [20–22]. Vitrification requires relatively high cooling and rewarming rates to outrun the rate of ice nucleation and growth [22–24].

Conventional inward cooling and rewarming by controlling the thermal environment of the organ container, results in a thermal gradient across the specimen [25–31]. This effect is only magnified by the relatively low thermal conductivity of CPAs, the high cooling and rewarming rates necessary for vitrification, and the size of the container. The increased thermal gradients increase the risk of structural damage due to thermomechanical stresses, with thermal expansion or contraction as its driving mechanism [32–34]. When that mechanical stress exceeds the strength of the material, structural damage follows, with fractures in the brittle vitrified material as the most noticeable outcome (*thermal stress* and *thermomechanical stress* are used interchangeably in this study). Thermal stresses due to temperature gradients can be reduced during the recovery of the specimen from cryogenic storage by using volumetric heating techniques [35–39]. This study focuses on the thermal stress developed during large-scale vitrification, when recovery from cryogenic storage is assisted by a special means of volumetric heating, known as *nanowarming* [35–38,40,41].

In broad terms, nanowarming is achieved by loading the specimen with a mixture of ferromagnetic nanoparticles and a CPA cocktail, and by exposing the specimen to an AC magnetic field thereby generating heating. The nanowarming effect investigated in this study targets silica-coated iron-oxide nanoparticles (sIONPs) [36,37,42], which are activated in the radio-frequency of 100–400 kHz [35,37,38]. These nanoparticles are washed out from the organ after nanowarming. For example, a washout efficiency of 93% was demonstrated experimentally for sIONPs on a rat heart model [35], where the remaining nanoparticles are found within the known tolerable concentration by the tissue. Of course, the washout efficiency is more broadly dependent on host of factors such as the nanoparticles size, shape, type, tissue model, and the washout protocol.

Recent experimental data and computational results suggest that the concentration of the nanoparticles is significantly affected by the organ geometry and its vasculature [35]. Since the specific nanoparticles tend to stay within the vasculature, the organ vasculature topology significantly affects the heating power distribution. In turn, this may affect the resulting thermal stresses. It has been further demonstrated that the container volume, geometry, and the nanoparticles concentration in the surrounding solution are key parameters that can assist in mitigating those adverse effects [43].

Specifically, this study focuses on the analysis of thermal stress in cryopreservation by vitrification of the heart, while exploring the effects of nanowarming-assisted recovery of the heart from cryogenic storage. This study aims at developing a new level of knowledge, following an experimental investigation of cryopreservation on a rat heart model [35], and theoretical thermal analyses of cryopreservation by vitrification on rat and human models [43]. Thermal stress modeling in this study is based on previous experimental investigations to explore the solid-mechanics response of the vitrified material [44–46], and theoretical studies to identify the effects leading to structural damage [32–34,47,48].

## Mathematical modeling

### Geometrical model

**Fig 1:**
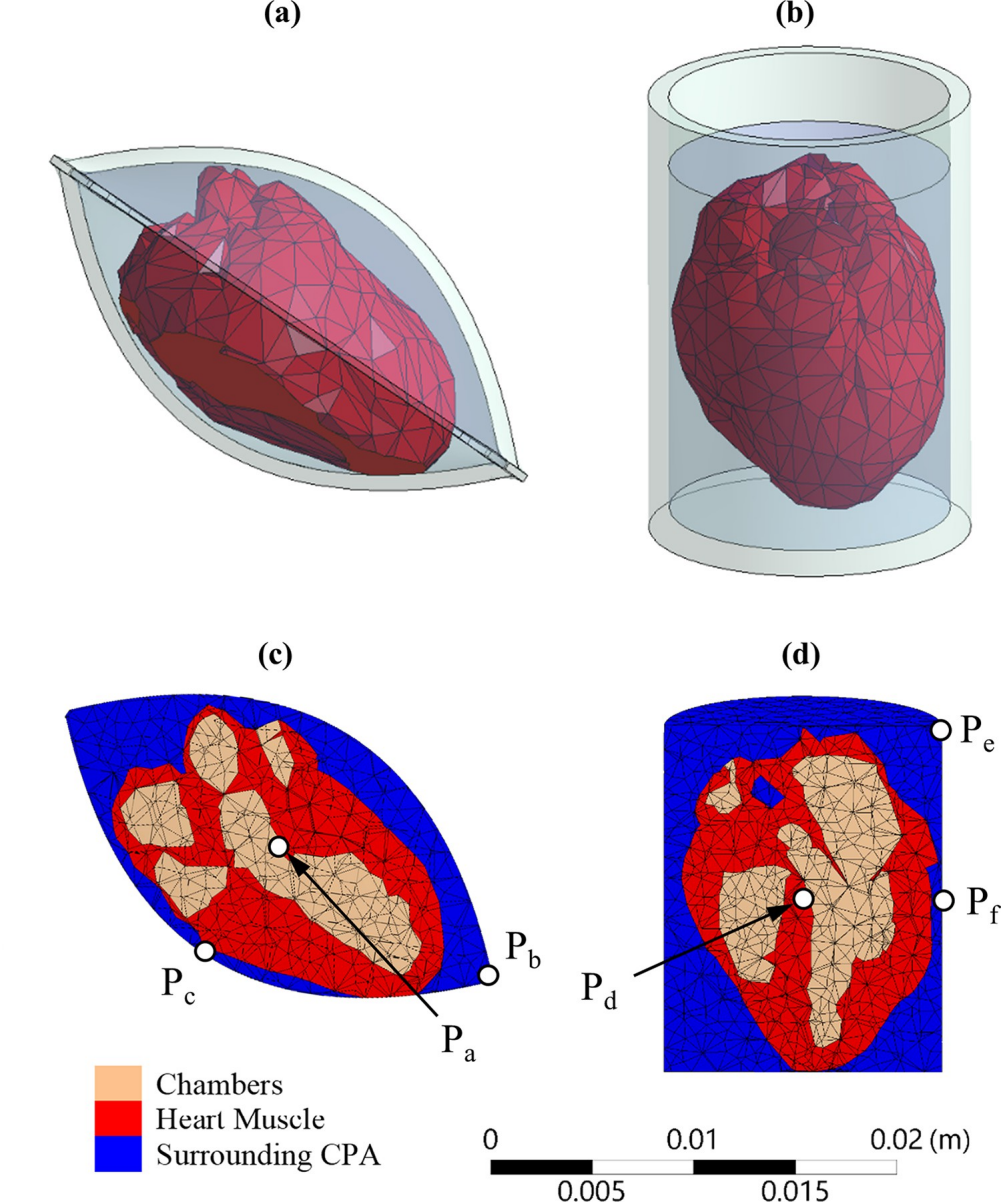
Illustrations of: (a) the heart model contained in a cryobag; (b) the heart model contained in a cylindrical container; (c) the cross section of the FEA mesh used for the heart-cryobag case, where P_a_ is the volumetric center of the domain, P_b_ is the center of the short cryobag edge, and P_c_ is the center of the bag’s surface; and (d) the cross section of the FEA mesh used for the heart-cylinder model, where P_d_ is the volumetric center of the domain, P_e_ is the base edge, and P_f_ is the mid-height on the container’s lateral surface.

The geometrical model used in this study has been presented recently [43] and is described here in brief for the completeness of presentation (Fig 1). The geometric model is based on MRI reconstruction of a healthy adult human heart, obtained from the Visible Heart Laboratory at the University of Minnesota. The geometric model was further refined to make it suitable for finite element analysis (FEM). For the comparative study of thermomechanical stress in the rat heart, a scale down similarity model was assumed, using the parameters listed in Table 1.

**Table 1:** Geometrical parameters of the heart model and its container (Fig 1).

Model		Volume, mL	Dimensions, mm
**Rat**	Heart	0.69	17.5 × 13 × 11
	Cryobag	2.2	23.4 × 17.5 × 12.5
	Cylinder ^†^	2.5	17.5 (L) × 13.5 (D); 22 (C)
		10	17.5 (L) × 27 (D); 22 (C)
**Human**	Heart	195	115 × 85 × 70
	Cryobag	618	154 × 115.5 × 85

† D and L represent the diameter and height of the CPA, respectively, while C represents the height of the container, which has a wall thickness of 1 mm.

Two container configurations are investigated in this study, an available pillow-shape cryobag [34] and a cylinder, as illustrated in Fig 1(A) and 1(B), respectively. The container dimensions were selected to closely match the heart volume with minimum surrounding solution, with dimensions listed in Table 1. The wall thickness for all containers and cryobags was selected to be 1 mm. The heart is assumed to be fully perfused with the cryoprotective agent (CPA), its chambers filled with CPA and the heart is fully immersed in CPA. However, the sIONP concentration may differ between those subdomains, as illustrated in Fig 1(C) and 1(D).

Three geometric reference points are selected in each model for thermal and stress analyses, as illustrated in Fig 1(C)–1(D), and addressed in the discussion section [34,47]. For the cryobag P_a_ is at the volumetric center, P_b_ is at the center of the shorter edge of the cryobag, and P_c_ is at the center of the bag’s surface. For the cylindrical container P_d_ is at the volumetric center of the container, P_e_ is at the base edge, and P_f_ is the mid height of the cylinder shell. The points P_b_, P_c_, P_e_, and P_f_ are selected at the interface between the container material and the contained CPA.

The container is assumed to be highly compliant for the purpose of solid mechanics analysis, and thus does not affect the mechanical stress developed due to the process of vitrification. This behavior is expected in common cryobag materials [32,34,47], while a confining effect of a stiffer container wall could further intensify the stress level [48]. Nonetheless, the container is rigid enough at higher temperatures to maintain its geometric shape without external support. Optimizing the container geometry and investigating the effects of various container materials is beyond the scope of the current study.

### Heat transfer model

Due to the high viscosity of the CPA [46], heat transfer within the container-organ-CPA system is assumed to be governed solely by conduction:

$$C\dot{T}=\nabla (k\nabla T)+\dot{q}$$ (1)

where *C* is the volumetric specific heat, *T* is temperature, *k* is thermal conductivity, the dot represents a time derivative, and $\dot{q}$ is the heat generated due to nanowarming:

$$\dot{q}=SAR\times C_{n}$$ (2)

where SAR is the specific absorption rate of the nanoparticles and *C*_*n*_ is the nanoparticles concentration in the CPA cocktail [42,49]. While this study focuses on lower cryogenic temperatures, when thermomechanical effects are significant, additional discussion about the effective thermal conductivity of CPAs at higher cryogenic temperatures has been discussed previously [46].

Continuity in temperature and heat flux is assumed on all internal boundaries, while a combined convective and radiative boundary condition is assumed between the outer surface of the container and the cooling environment:

$$-k_{j}\frac{{\partial T}_{j}}{\partial \hat{n}}=U(T_{j}-T_{c})$$ (3)

where $\hat{n}$ is the normal to the container’s outer surface and *U* is the overall heat transfer coefficient, combining effects of convection and thermal radiation, and the indices *j* and *c* represent the container’s external surface and the cooling environment [46].

### Solid mechanics model

The CPA solution is modeled as a Maxwell fluid [50], where the viscosity increases exponentially with the decreasing temperature by fifteen orders of magnitude, Table 2. Here, the total strain rate is calculated by:

$${\dot{\varepsilon }}_{total}={{\dot{\varepsilon }}_{elastic}+\dot{\varepsilon }}_{creep}+{\dot{\varepsilon }}_{thermal}$$ (4)

where the elastic, creep, and thermal strain rates are calculated by:

$${\dot{\varepsilon }}_{elastic}=\frac{1}{E}[\left(1+\nu )\dot{\sigma }-\upsilon I∙tr(\dot{\sigma }\right)]$$ (5)

$${\dot{\varepsilon }}_{creep}=\frac{S}{2\eta }$$ (6)

$${\dot{\varepsilon }}_{thermal}=\beta \;\dot{T}\;I$$ (7)

where *E* is the Young’s modulus, *ν* is the Poisson ratio, *σ* is the stress, ***I*** is the identity tensor, *tr* is the trace of the stress tensor, ***S*** is the deviatoric stress tensor, *η* is the viscosity, and *β* is the thermal expansion coefficient. In order to reduce computational costs, the container material has not been modeled in the solid mechanics model, which, in practice, would yield the same results as in the case of a highly compliant container [34,51]. In addition, continuity in displacement is assumed on all internal interfaces. Recall that a highly compliant container is assumed, which means zero normal stress on all outer surfaces of the domain.

**Table 2:** Material properties used for modeling in the current study.

Property	Material	Value (temperature in°C)
**Specific heat,** ***C***_***p***_ **(*J*/*kg*°C)**	VS55	7.60T+2402.3−150≤T<−120 $\begin{array}{l} 545.24T+66747-120\leq T\leq -119\\ 0.039T^{2}+11.73T+2678.8-119<T\leq 10 \end{array}$ [37]
	VS55+0.6M Sucrose	$0.04T^{2}+19.4T+2688.7-150\leq T\leq -118$ $-6.11T^{2}-1238T-60074-118<T\leq -104$ $0.01T^{2}+3.96T+2803-104<T\leq 10$ [52]
	Polyethylene	$1031+3.57\times T-0.014\times T^{2}-6.1710\times 10^{-5}\times T^{3}$ [53]
**Melting temperature, *T***_***m***_ **(°C)**	VS55	-38 [37]
**Critical cooling rate, *CCR*(°C/*min*)**	VS55	2.5 [37]
	VS55+0.6M Sucrose	<1 [52]
**Critical rewarming rate, *CWR*(°C/*min*)**	VS55	50 [37]
	VS55+0.6M Sucrose	<1 [52]
**Glass transition temperature, *T***_***g***_ **(°C)**	VS55	-123 [37]
	VS55+0.6M Sucrose	-115 [52]
**Density, *ρ* (*kg*/*m*** ^**3**^ **)**	VS55	1078−0.43*T* [54]
	Polyethylene	$931-0.49\times T-0.00091{\times T}^{2}$ [55]
**Thermal conductivity,** ***k* (*W*/*m*°C)**	VS55+sIONP	$8.16\times 10^{-4}T+4.40\times 10^{-1}-180\leq T\leq -116$[56] $-1\times 10^{-3}T+2.34\times 10^{-1}-116<T<-96$[56] $4.59\times 10^{-4}T+3.74\times 10^{-1}-96\leq T\leq -77$[56] $5\times 10^{-4}T+3.74\times 10^{-1}-77<T<-34$[56] $4.59\times 10^{-4}T+3.74\times 10^{-1}-34\leq T$[56]
	Polyethylene	$0.39+5.2\times 10^{-4}\times T$ [57]
**Viscosity, η (Pa-s)**	VS55	$1.21\times 10^{4}-100\leq T$ [50] $4.2783\times 10^{-23}e^{-0.6091T}-140\leq T<-100$ [50] $4.63\times 10^{14}T\leq -140$ [50]
	VS55+0.6M Sucrose	$1.21\times 10^{4}-92\leq T$ $4.2783\times 10^{-23}e^{-0.6091T}-132\leq T<-92$ $4.63\times 10^{14}T\leq -132$ (Assumed)
**Thermal Expansion, α (°C** ^**−1**^ **)**	VS55	1.1 × 10−4 [47]
**Young’s Modulus, E (Pa)**	VS55	800 × 10^6^ [58,59]
**Poisson’s Ratio, ν**	VS55	0.25 [47]

### Physical properties and operational parameters

Consistent with a recent experimental investigation [35], the analysis in this study focuses on the material properties of VS55 as a CPA solution when loaded with silica-coated iron nanoparticles (sIONPs). VS55 is a cocktail of 3.1 M dimethyl sulfoxide (DMSO), 2.2 M propylene glycol, and 3.1 M formamide. Additionally, VS55 mixed with 0.6 M sucrose as a synthetic ice modulator [60] loaded with sIONPs is considered, due to sucrose’s favorable effect of inhibiting ice nucleation and growth [52]. While the specific values of the critical cooling rate (CCR) and the critical warming rate (CWR) of VS55 mixed with 0.6 M sucrose remain undetermined, they are below 1°C/min based on a recent differential scanning calorimetry study [52]. While the melting temperature of VS55 is -38°C (which is also the upper temperature boundary for heterogeneous nucleation), the melting temperature of the cocktail VS55+0.6M sucrose also remains unknown. A previous differential scanning calorimetry study on the closely related CPA cocktail DP6 (3 M DMSO and 3 M propylene glycol) suggested that adding 0.6 M sucrose may lower the melting temperature (and hence the upper boundary of heterogenous nucleation) by more than 10°C by extrapolation, but specific measurements have not been obtainable in that study due to experimentation limitations and the extremely low nucleation rate of the material [61].

The specific heat generation rate for nanowarming, Eq (2), is modeled based on recent measurements of VS55 mixed with sIONPs (EMG-308 Ferrotec) when excited at a field strength of 62 kA/m and frequency of 185 kHz [35]. It follows that the SAR is approximated as linearly decreasing from 691 W/g Fe at -80°C to 415 W/g Fe at -20°C, respectively [40]. A follow-up experimental study (unpublished) suggests the same linear SAR decrease up to 0°C. In the absence of additional SAR experimental data, the SAR values are assumed to remain constant below -80°C and above 0°C [43].

During cooling, an overall heat transfer coefficient (*U* in Eq 3) of 80 W/m^2^K is assumed for the rat heart model based on least-square parametric estimation from experimental data [35], while a 350 W/m^2^K value is assumed for the human heart model, which was found necessary to ensure successful vitrification [43]. Further note that the latter value was found experimentally as the upper limit for heat transfer rate in a commercial controlled-rate cooler [46]. During rewarming, the overall heat transfer coefficient was investigated within the range of 15 W/m^2^K and 350W/m^2^-°C to identify the best rewarming conditions, where the lower value is representative of free convection and radiation [35] while the upper value is representative of the maximal convective conditions feasible in the experimental system.

Table 2 lists all other physical properties used in the current study. In the absence of physical properties of the CPA-loaded tissue, due to the high CPA concentration in the tissue, and consistent with previous studies [62], the same CPA properties are taken for both the heart model and the surrounding solution. Previous studies have shown that the mechanical response of vitrified tissues loaded with CPA solution at lower temperatures is dominated by the CPA solution [54,58,63]. In the context of CPA distribution in the specimen, previous experimental results based on micro-CT imaging of the rat heart demonstrated 100% CPA solution loading in the ventricles, and up to 86% CPA solution loading of the heart on average [35]. In this context, 100% loading means that the entire respective volume is filled with the CPA solution with no dilution. Furthermore, Peyridieu et al. [64] have demonstrated experimentally that the CCR and the CWR in CPA-loaded tissues may be dramatically lower than those required for pure CPA. With these experimental observations in mind, the use of the original CPA solution’s physical properties for the CPA-loaded tissue is considered a good approximation in the current study, with a somewhat conservative CCR and CWR criteria to ensure vitrification based on computer modeling.

Due to the limited availability of relevant CPA properties and due to its high relevancy to vitrification, the CPA cocktail VS55 is used as a model for material properties when specific material properties of VS55 mixed with sucrose are unavailable. A previous study concluded that the viscosity value of a CPA solution is not particularly important as long as the annealing temperature is appropriately shifted with respect to the glass transition temperature [47]. Therefore, in the absence of viscosity values for VS55+0.6 M sucrose, the viscosity values of VS55 are used by appropriately shifting them with respect to the difference between the glass transition temperatures of both the solutions (8°C in this case).

### Cryogenic protocol

*Rat model*: Replicated from a previous experimental investigation [35] and consistent with the thermal analysis presented previously [43], the cooling portion of the thermal protocol was kept identical for all cases studied on the rat heart model: (a) initial cooling of the chamber at a constant rate of 40°C/min from an initial temperature of -20°C down to -122°C, (b) temperature hold at -122°C until the specimen reaches thermal equilibrium, (c) further cooling at a constant rate of 40°C/min to the cryogenic storage temperature of -150°C, and (d) indefinite temperature hold at cryogenic storage. This protocol resulted in the complete vitrification of the rat heart muscle and surrounding solution [35].

Based on prior experiments of the rat heart model [35], the analysis focuses on the CPA cocktail VS55. An sIONP concentration of 10 mg Fe/ml was selected for the heart chambers, which resulted in 1.47 mg Fe/ml in the heart muscle due to the underlying effects governing nanoparticle loading [35]. Successful nanowarming cases identified in the previous thermal analysis [43] were now investigated for thermomechanical stress effects, as listed in Table 3. In addition, a reference case was investigated in this study (Case I in Table 3), subject to a uniform sIONP concentration of 10 mg Fe/mL throughout the CPA solution, heart muscle, and the chambers of the heart. This concentration would result in the fastest uniform rewarming throughout the domain. Table 3 also lists variable boundary conditions for rewarming, which were experimented with to achieve the best rewarming outcomes as discussed below.

**Table 3:** Boundary conditions and nanoparticles concentration in the surrounding solution for selected rat heart model cases [43], where the time, *t*, is measured in seconds.

Case	sIONP concentration, mg Fe/mL			Boundary condition during rewarming	Container geometry
	Chambers	Heart Muscle	Surrounding solution		
**I**	10	10	10	Adiabatic	Cryobag, Cylinder
**II**		1.47	7.5	Adiabatic	Cryobag, Cylinder
**III**			5	Convection *h*_*eff*_ = 15W/m^2^K *T*_*chamber*_ = 22°C	Cryobag
**IV**			3.75	Convection *h*_*eff*_ = 80 W/m^2^K *T*_*chamber*_ = -150°C– 100*t*	

All the cases investigated resulted in successful nanowarming from thermal analysis consideration, which satisfied the following criteria: (i) the rewarming rate anywhere in the specimen and at any point in time during rewarming exceeded the critical rewarming rate of the CPA solution used, and (ii) the temperature distribution at the end of rewarming did not fall below -35°C (3°C safety margin above melting temperature) or above 0°C. While the reasoning for those temperature thresholds is discussed in detail in the preceding thermal analysis study [43], in broad terms, the -38°C value represents the upper limit for heterogeneous nucleation (melting temperature) of the CPAs investigated [37], while the 0°C represents the upper limit for pure water crystallization.

*Human Model*: While the above analysis of the rat heart is based on VS55, its critical cooling and rewarming rates in combination with the slower thermal response of the larger human heart to the surroundings temperature make that CPA cocktail impractical [43]. Therefore, the analysis of the human heart in the current study focuses on the mixture of VS55 with 0.6M sucrose as a SIM, which yields sufficiently lower critical cooling and rewarming rates (Table 2), as demonstrated previously [43]. Consistently, the solid mechanics analysis in the current study uses the same thermal protocol investigated there [43]: (a) initial cooling of the chamber at a constant rate of 40°C/min from an initial temperature of -30°C down to -118°C (3°C below *T*_*g*_ of VS55+0.6M sucrose [52]); (b) temperature hold at -118°C until the specimen reaches thermal equilibrium to facilitate stress relaxation; (c) further cooling at a rate of 1°C/min to the cryogenic storage temperature of -150°C; (d) indefinite temperature hold at cryogenic storage; (e) slow convective rewarming from a storage temperature of -150°C up to -116°C (1°C below *T*_*g*_), subject to a convective boundary condition while the chamber rewarms at 1°C/min; (f) temperature hold when the boundary temperature reaches -116°C to achieve thermal equilibrium; and (g) nanowarming combined with a convective boundary condition, while the chamber temperature increases at a rate of 5°C/min. Thermal equilibrium is assumed at the respective temperature-hold steps when the maximum temperature variation across the heart is less than 0.01°C. While the thermal protocol is being applied for thermal constraints, it is noted that, contrary to common perception, the maximum stress during vitrification does not necessarily increase with increasing size of the specimen [51].

An overall heat transfer coefficient of 350 W/m^2^°C was selected for the human heart modeling, being the highest coefficient achievable in a controlled-rate cooler of compatible size, in order to minimize the temperature difference between the specimen and the changing environment. Additionally, the ratio of nanoparticle concentration between the various subdomains was kept 10:1.47:3.75 between the chambers, muscle, and surroundings, respectively, from practical and thermal considerations [43]. Based on that study, an example of thermally successful human heart nanowarming suggests the following volumetric heat generation in the heart chambers: a constant value of 423 W/m^3^ below -80°C, followed by a linearly dependent value between 423 W/m^3^ at -80°C and 260 W/m^3^ at -20°C, while maintaining the above ratio of 10:1.47:3.75 between the chambers, muscle, and surroundings at all times.

### Computational framework

The current modeling is based on the underlying assumption that heat generation due to mechanics effects are negligible, which leads to a unidirectionally coupled thermo-mechanical analysis. It follows that the transient thermal field affects a mechanical response in the system, but mechanical effects do not give rise to heat generation and thereby do not affect heat flow [32,34,47]. The FEA commercial code ANSYS was used for the coupled thermal and mechanical modeling. In total between 11,600 and 69,000 elements were selected for the various geometries described above, based on the convergence analyses. A remote displacement boundary condition was applied to the model to prevent rigid body motion in space.

## Results and discussion

The discussion herein starts with an idealized case of nanowarming in a rat heart model as a reference, Case I in Table 3, and then advances to more realistic cases of nanowarming distribution. Finally, the discussion advances to the analysis of the human heart model.

### Rat heart model

#### Case I: Uniform nanoparticles distribution

**Fig 2:**
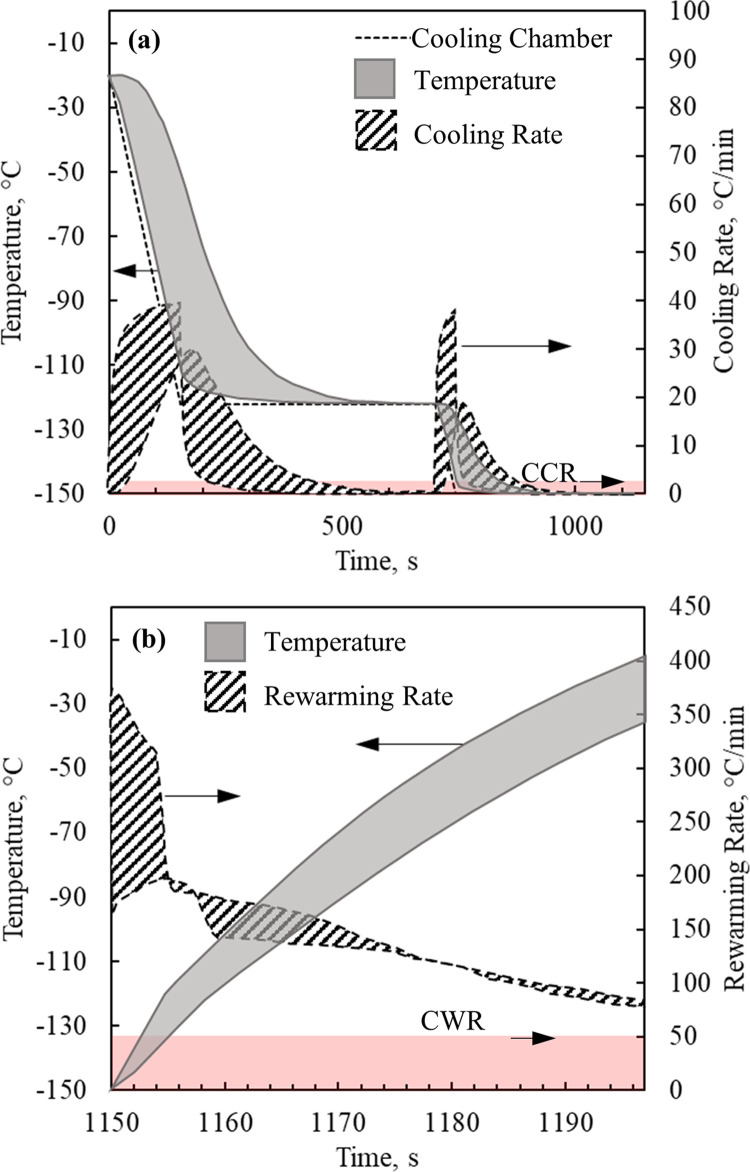
Thermal history results for the benchmark case of a rat heart model contained in a cryobag, Case I (Table 3), where results refer to the heart muscle during (a) cooling, and (b) rewarming. Note: During cooling, the minimum cooling rate dips below the CCR. This rate corresponds to the region that has cooled down the fastest and is already at equilibrium with the chamber.

P_c_P_a_Fig 2 displays the thermal history during cooling and rewarming in Case I, which corresponds to an ideal case of vitrification using VS55 as a CPA and uniformly distributed sIONP at a concentration of 10 mg Fe/mL across all the subdomains of the model: cryobag, heart muscle, and chambers. Fig 2(A) displays the time-dependent ranges of cooling rate and temperature within the rat model. As can be seen from Fig 2(A), the cooling rates exceed the CCR required to avoid crystallization in the temperature range of -35°C to -80°C, where the probability of crystal growth decreases exponentially with temperature and is considered very low below -90°C for all practical matters [65]. It should be noted that in Fig 2(A) the cooling rate dips below the CCR. This corresponds to the region which has cooled down the fastest and now is at equilibrium with the cooling chamber. It is also acknowledged that while the ice growth rate is very low in such low temperatures, the ice nucleation rate may be significant [24]. Notably, the cooling rate variation across the domain reaches a very narrow range each time that the chamber cooling rate changes.

Fig 2(B) displays the thermal history during the nanowarming, where the instantaneous temperature variation increases with the progression of rewarming. Nanowarming is terminated when the lowest temperature anywhere in the heart muscle exceeds the melting point by a 3°C safety margin, which is -38°C for VS55. As can be seen from Fig 2(B), the rewarming rate anywhere in the domain in Case I exceeds the CWR, which is favorable for preventing rewarming-phase crystallization. Nonetheless, the increasing temperature variation during nanowarming may result in significant thermomechanical stresses, which is the focus of the current study.

**Fig 3:**
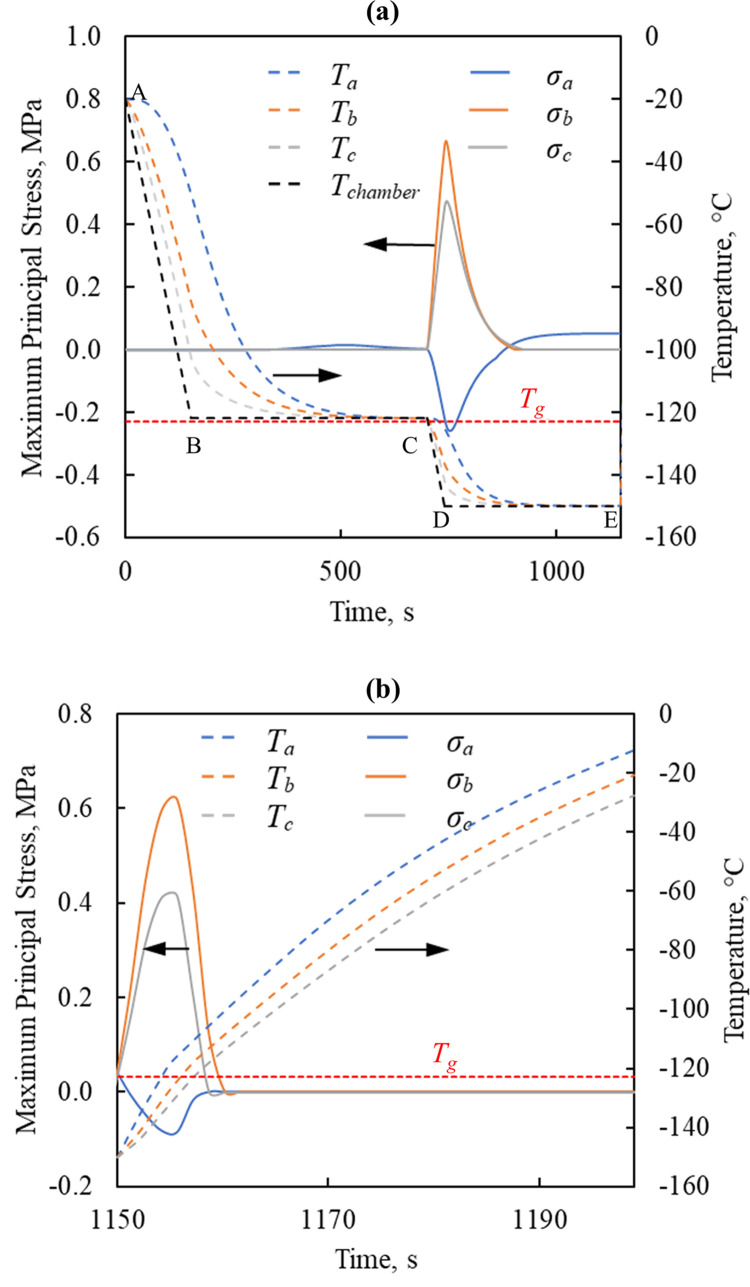
Thermal and maximum major principal stress history in Case I (Table 3) for the rat heart model when contained in a cryobag model (Fig 1) during (a) cooling and (b) rewarming.

Fig 3 displays the thermal history and major principal stress history at the three representative points in the cryobag illustrated in Fig 1(C). Based on the general convention, a positive value of stress corresponds to tension, while a negative value corresponds to compression. It is emphasized that the principal stresses are the normal stress calculated at such an angle that shear stresses become zero, which is considered detrimental in brittle materials. The principal stress with the maximum value is defined herein as the *major principal stress* (or the *first principal stress*), *σ*_*P1*_, where its orientation may change over time and across the domain. Being a brittle material, the vitrified CPA is much more likely to fail in tension rather than in compression, where the magnitude of the compressive strength is several folds higher than the tensile strength. The strength-to-fracture under tension of vitrified CPA is of the order of 3.2 MPa [33], although higher strain rates will lead to structural damage at lower stress levels. Further note, that significant stress may prevail above the glass transition temperature and below the so-called *set temperature* [33]. While the difference between the glass transition temperature and the set temperature is cooling-rate or rewarming-rate dependent, its magnitude may exceed 10°C [33]. This means that structural damage may prevail even above the glass transition temperature.

The stress analysis during the cooling portion of a cryogenic protocol has been presented in detail previously [34,47,51] and is presented here in brief for the completeness of the presentation. It can be seen from Fig 3(A) that no significant stresses develop during the initial cooling segment (A-B). Despite the significant temperature variation and gradients during that fast initial cooling stage, stress relaxation occurs almost instantaneously due to the low viscosity of the CPA in the relevant temperatures, and the cooled material practically remains stress free down to point C in the figure. The stress relaxation segment B-C is designed to allow for thermal equilibration while the material is free to flow. Once the cooling commences in segment C-D, the vitrifying material starts to behave like a solid, and significant stresses may develop. Due to the exponentially increasing viscosity with the decreasing temperature, the vitrified material behaves like a linear-elastic solid below the glass transition temperature in any practical time scale. Due to the inwards cooling process in segment C-D, the center of the domain experiences maximal principal stress in compression, while the outer portion of the domain experience it in tension. This trend reverses itself during cryogenic storage, segment D-E, where the major principal stress in tension is now found at the center of the domain. However, since the vitrified material now behaves as a linear-elastic material, that stress cannot dissipate, and the outcome is commonly referred to as *residual stress*.

Significant stresses are observed during the onset of rewarming only when the temperatures are below glass transition, Fig 3(B). Unlike during traditional convective heating at the outer surface, the center of the container rewarms at a much faster rate due to the presence of nanowarmers. While one could expect a uniform instantaneous temperature distribution during rewarming subject to adiabatic external boundary conditions, the container material also plays a role in the thermal process, which results in temperature gradients. As observed previously [32], Case I leads to reduced stress at the center of the domain during rewarming, as now the developing stress counteracts the residual stress. Reducing the nanoparticle concentration uniformly throughout the domain further reduces *σ*_*MP*,*max*_ in tension. For example, lowering the sIONP concentration in Case I from 10 Fe/mL to 7.5 mg Fe/mL and 5 Fe/mL, while keeping all other parameters unchanged, reduces *σ*_*MP*,*max*_ in tension from 0.67 MPa to 0.61 MPa and 0.5 MPa, respectively.

While the above discussion is focused on the cryobag geometry, the stress development and distribution in other container geometries display similar trends. Table 4 lists values for *σ*_*MP*,*max*_ in various container geometries for Case I. For the cylindrical containers, the *σ*_*MP*,*max*_ in tension during cooling occurs at point P_f_, the maximum principal stress during storage occurs at point P_d_, and the *σ*_*MP*,*max*_ in tension during nanowarming occurs at point P_e_ (Fig 1).

**Table 4:** Maximum principal stresses in various stages of the protocol for reference Case I (rat heart model), where the smaller cylinder is selected to tight fit to the rat heart, while large cylinder is double the diameter and quadruple the volume container of the smaller container for practical reasons.

Container	Cooling		Storage, MPa		Rewarming, MPa	
	Tension, MPa	Compression, MPa	Tension, MPa	Compression, MPa	Tension, MPa	Compression, MPa
**Cryobag**	0.75	0.5	0.05	0.03	0.67	0.2
**Small Cylinder** † **17.5 mm (L) × 13.5 mm (D); 22 mm (C)**	0.81	0.7	0.06	0.02	1.1	0.25
**Large Cylinder** † **17.5 mm (L) × 27 mm (D); 22 mm (C)**	1.1	0.78	0.1	0.05	1.3	0.24

† D and L represent the diameter and height of the CPA, respectively, while C represents the height of the container.

#### Non-uniform concentration of nanoparticles

A key effect in the application of nanowarming is the variation of nanoparticle concentration across the domain, either by design or due to physical limitations. For example, it is conceivable to fill the heart chambers with a CPA solution containing a different concentration of nanoparticles than that in the solution surrounding the organ thereby affecting the thermal history across the organ. As another example, the physical limitations on nanoparticle permeation may limit their loading beyond that organ vasculature [35]. Furthermore, it has been demonstrated in a previous study [43] that balancing between the boundary conditions and the sIONP concentration in the surrounding solution can yield improved thermal performance. Consistently, Table 3 displays three additional rewarming cases investigated in this study, while the cooling portion of the protocol has been kept similar to the reference Case I.

**Fig 4:**
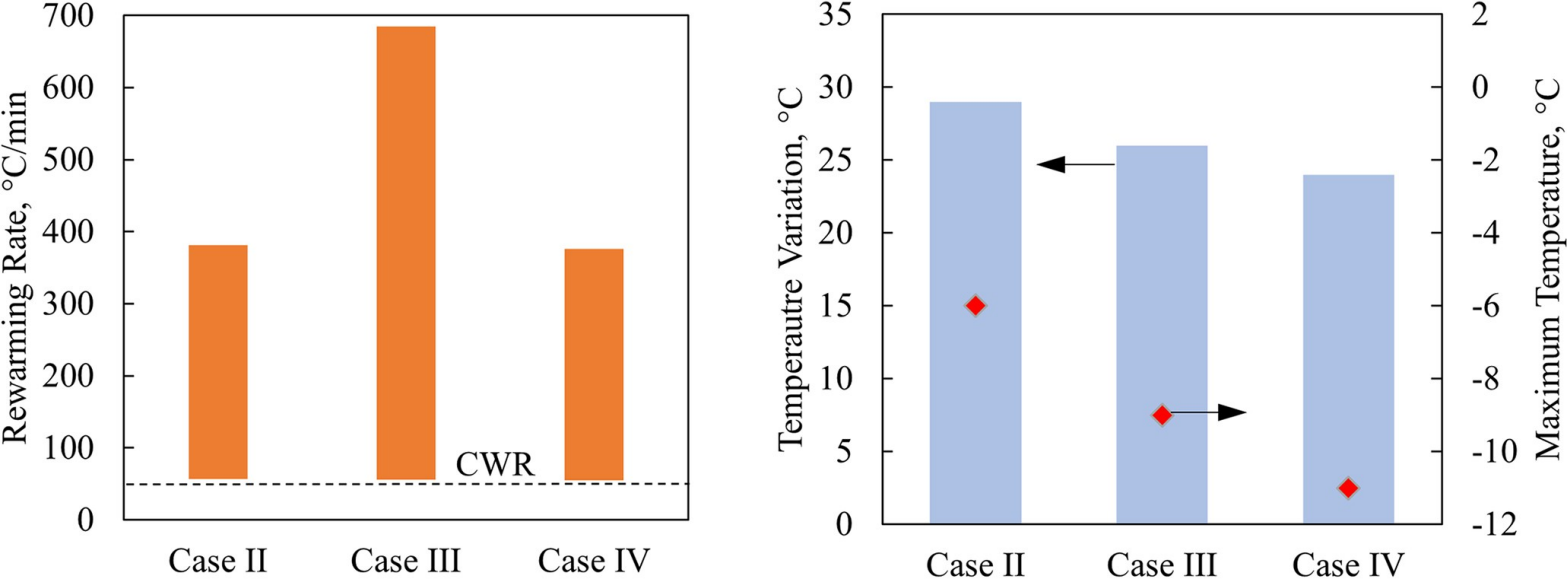
Comparative thermal results for the rat heart muscle contained in a cryobag in Cases II-IV: (a) rewarming rates range, and (b) temperature variation at the end of nanowarming, when the lowest temperature in the heart surpasses -35°C (bars), and the maximum temperature at that instant (red diamonds).

**Fig 5:**
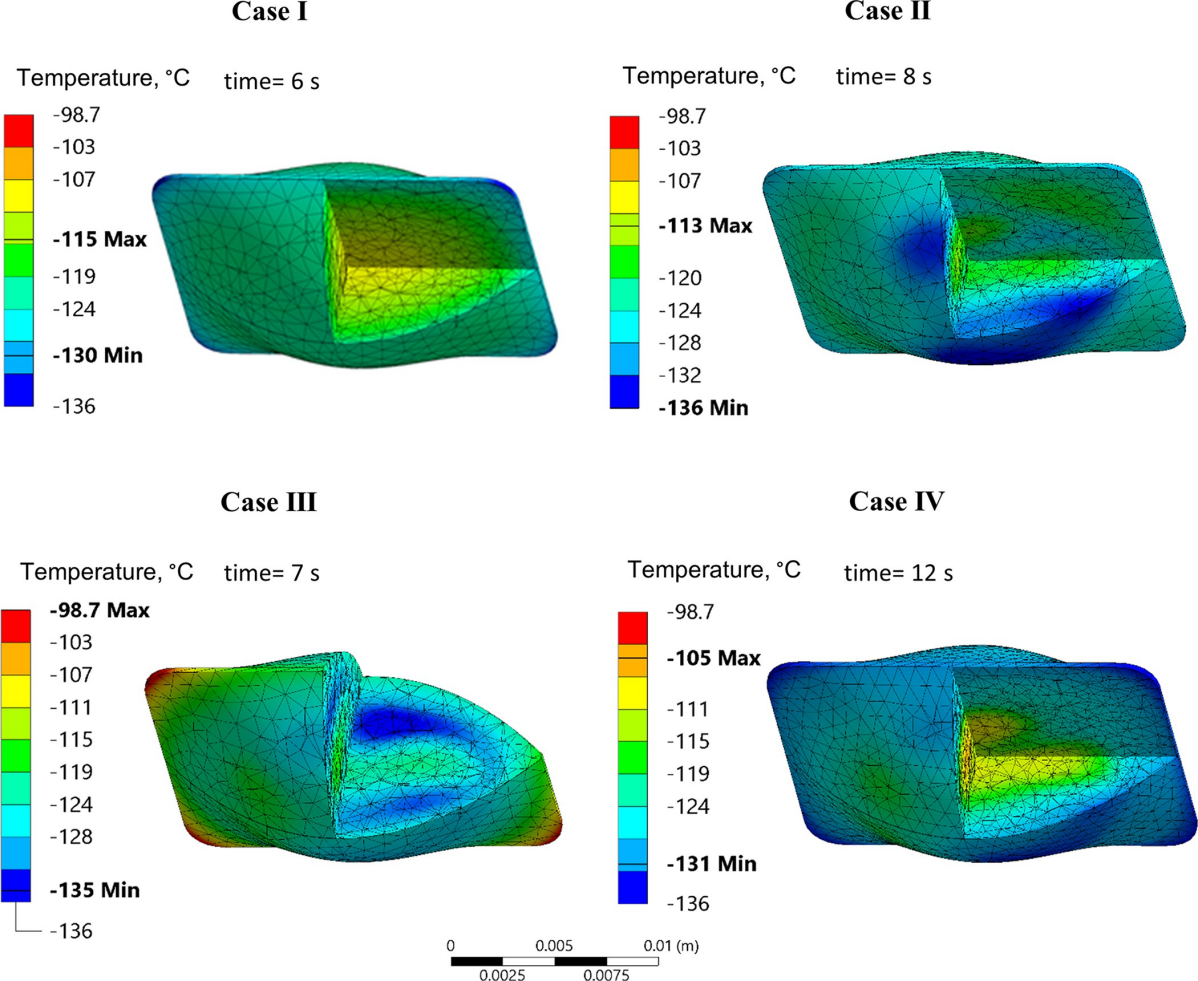
Temperature distribution in the rat heart model contained in a cryobag for Cases I-IV at an instance in time when maximum principle tensile stress occurs during rewarming. Time displayed is the time from the beginning of rewarming.

It can be seen from Fig 4(A) that all the variable nanoparticle concentration cases resulted in rewarming rates exceeding the CWR of VS55 throughout the process. Fig 4(B) displays (i) the temperature variation in the cryobag model at the end of nanowarming when the lowest temperature in the heart surpasses -35°C, and (ii) the maximum temperature observed in the heart at that instant. While Fig 4 displays the overall range of temperatures and rewarming rates, the spatial points at which the maximum temperature and minimum rewarming rate vary along the rewarming process, as demonstrated in Fig 5. Although not explicitly observed from Figs 4 and 5, the maximum temperature variation is typically found towards the end of rewarming. This may have further implications on the CPA toxicity effect while waiting for final temperature equilibration to facilitate CPA unloading. While this study is not aimed at analyzing toxicity effects, the last comment is meant to alert to another dimension in the space of nanowarming modeling and optimization.

**Fig 6:**
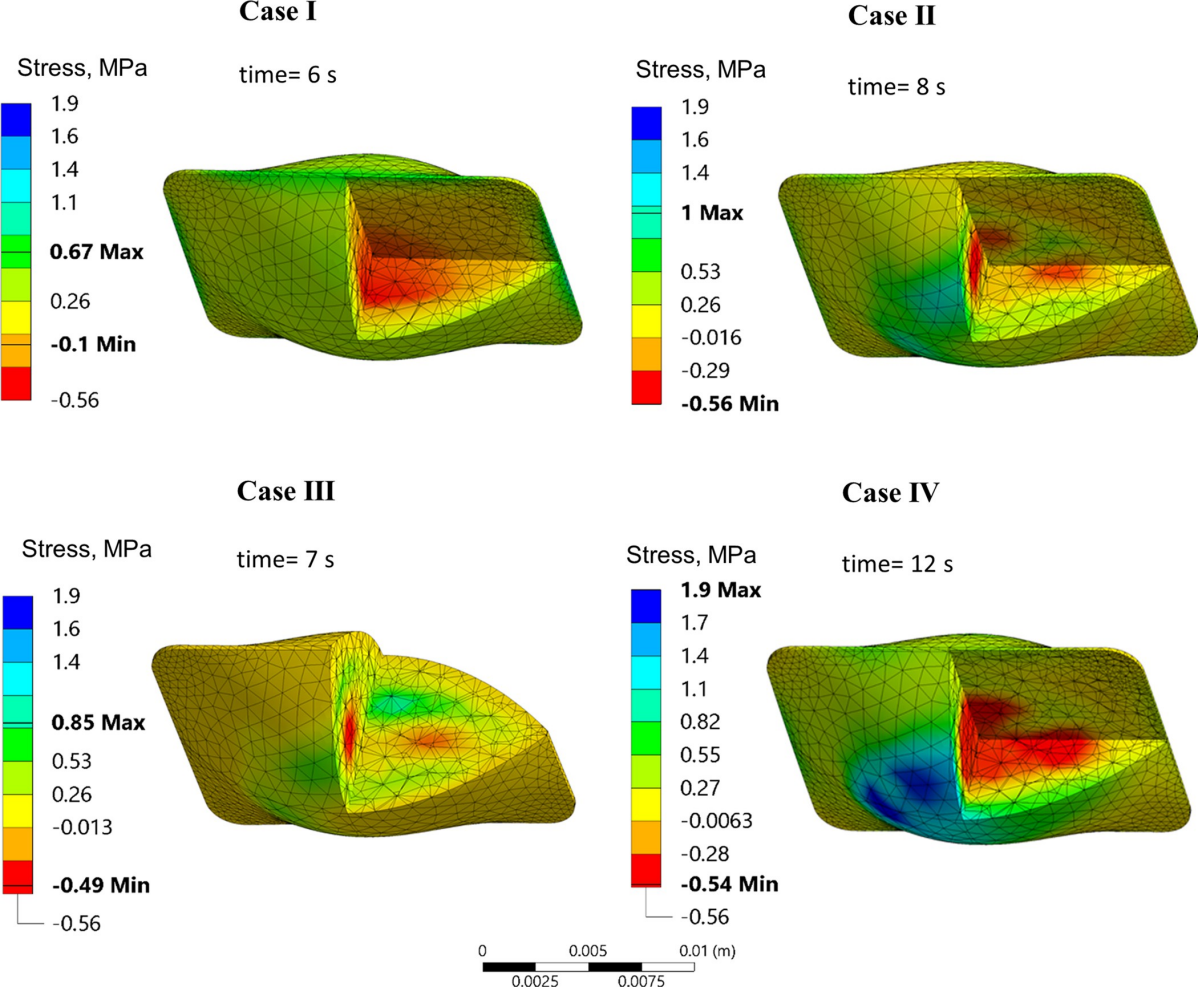
Major principal stress in the rat heart model contained in a cryobag, at the point in which the principle tensile stress reaches its maximum value. Time displayed is the time from the beginning of rewarming.

Fig 6 displays *σ*_*MP*_ distribution for Cases I-IV in the cryobag geometry, at the time in which the maximum *σ*_*MP*,*max*_ in tension occurs during rewarming (the time for that to be observed varies among cases). Three general observations can be made from Figs 5 and 6: (i) non-uniform sIONP concentrations resulted in higher stresses during rewarming in Case II-IV as compared with the uniform nanoparticle distribution in reference Case I; (ii) the highest magnitude of stress occurs when the temperature in the entire domain is between the storage temperature (-150°C in this case) and about -100°C, above which the decreasing viscosity of the solution allows for CPA flow to facilitate stress dissipation; and (iii) throughout rewarming, the regions that rewarm more rapidly (for example, the chambers of the heart) undergo compressive stresses, while the slower rewarming regions (for example, the heart myocardium) undergo tension. This observation is consistent with the concentration of sIONPs, whereas the chambers of the heart have the highest sIONP concentration while the heart muscle has the lowest. The effects of stress decay above -100°C, can be observed in more detail in Case I for example in Fig 3(B).

While the above general observations remain the same in all the cases investigated, the location and direction of *σ*_*MP*,*max*_ varies among the cases presented in Fig 6: the epithelium of the heart in contact with the container in Cases II and IV, and in the myocardium of in Case III. Observing the temperature distribution and the corresponding stresses developed, the regions susceptible to higher tensile stresses are regions that have a higher thermal gradient, which is correlated with variations in nanoparticle concentration. These results suggest that a mismatch between the rewarming rates on the surface of the specimen in contact with the container, the container itself, and the chambers of the heart, exacerbate the maximum stresses observed during rewarming. A remedy to reduce the thermomechanical stress can be found by matching the convective boundary condition with the internal heat generation rate of the CPA, as demonstrated in Case III [43].

The dramatic effect of matching the boundary conditions with the internal heat generation can be observed from the maximum tensile stress values in Table 5. Also shown there is the correlation between the maximum stress and the fastest and slowest rewarming rates in the specimen. For reference, the maximum values of *σ*_*MP*_ during cooling is 0.75 MPa in tension and 0.5 MPa in compression. The residual *σ*_*MP*_ during storage varies between +0.05MPa and -0.03MPa.

**Table 5:** Maximum principal stresses during cryobag rewarming in Cases I-IV for the rat heart, subject to a common thermal history and stress distribution during cooling and storage, where the rewarming rate is calculated as the localized time average between the onset of rewarming and the instant at which the maximum stress occurs.

Case	Maximum Principal Stress, MPa		Rewarming Rate,°C/min		
	Tension	Compression	Fastest in the domain	Slowest in the domain	Average
**I**	0.67	0.2	374	205	340
**II**	1.0	1.0	271	105	198
**III**	0.85	1.0	410	137	213
**IV**	1.96	1.3	222	97	140

#### The container effect

While the current study focuses on the cryobag as a choice of practice, a cylindrical container could also be a viable practical alternative. Consistently, two additional container geometries are considered in this study: a smaller cylinder that tightly fits the heart, and a bigger cylinder essentially corresponds to double the diameter and quadruple the volume container for reference, Table 4 and Fig 1. Consistent with the thermal study [43], the cryobag subject to the thermal conditions in Case II, Table 3, is associated with improved thermal performance, and hence it is selected here for the comparison between the cylinder and the cryobag geometry.

**Fig 7:**
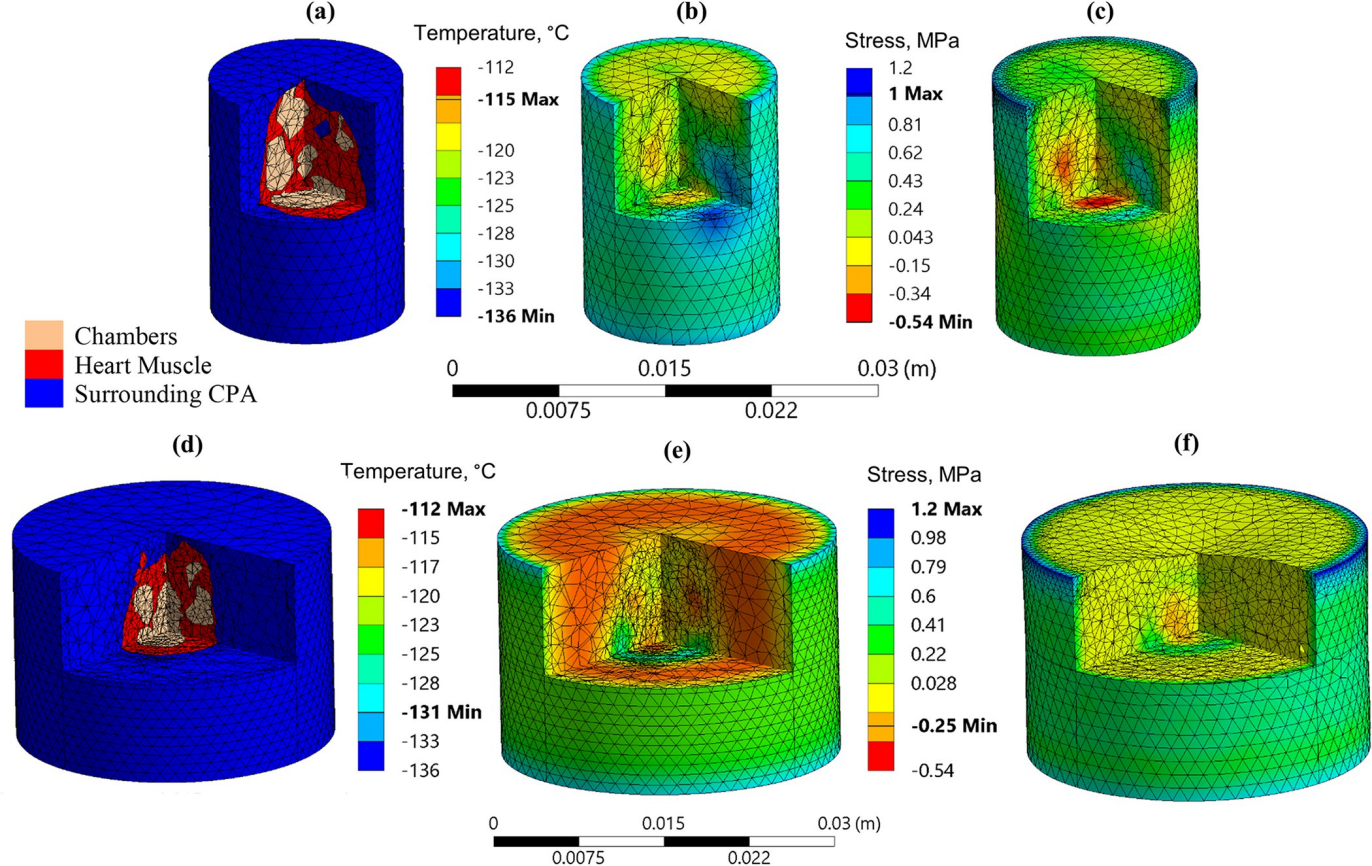
FEA mesh, major principal stress, and temperature distribution in the cylindrical containers for the rat heart model in Case II: (a)-(c) correspond to the smaller container, while (d)-(f) correspond to the larger cylinder, Table 1. The temperature distribution and stresses are plotted at an instance in which the maximum major principal stress occurs during rewarming.

The *σ*_*MP*,*max*_ values in the three containers during rewarming are listed in Table 6. It can be observed that while the *σ*_*MP*,*max*_ in tension was similar for the cryobag and the smaller cylinder, it increases by 20% for the larger cylinder. It can be observed from Fig 7, that the maximum stress in tension is at the edge of the top base of the cylinder, where the CPA solution is in contact with the container wall, regardless of the cylindrical container size. Recall that the current study focuses on a highly compliant container wall, while thermal expansion mismatch between the wall and the CPA could increase the localized stress there dramatically [33,34,51]. Nonetheless, while the stress caused by the container walls is not investigated in the current solid mechanics study, the effect of thermal gradient and temperature distribution developed due to the container geometry, material, and boundary conditions are taken into consideration. In this case, the extended portion of the cylindrical container wall extends above the CPA solution (Fig 1(B)) and acts as an extended surface to heat dissipation to the chamber (i.e., a *thermal fin*), while heat transfer to the chamber from the outer surface of the container is blocked (i.e., an adiabatic condition). This effect increases the temperature gradients there and thereby leading to a stress concentration.

**Table 6:** Maximum principal stresses in various geometries for Case II for a rat heart model, where the thermal history and container geometries remained unchanged from Case I (Table 4), and where the rewarming rate is calculated as the localized time average between the onset of rewarming and the instance at which the maximum stress occurs.

Container	Maximum Principal Stress, MPa		Rewarming Rate,°C/min		
	Tension	Compression	Fastest in the domain	Slowest in the domain	Domain average
**Cryobag**	1.0	1.0	271	105	198
**Small Cylinder**	1.0	0.84	296	125	201
**Large Cylinder**	1.2	0.53	270	147	156

These results suggest that apart from the sIONP concentration, shape, volume of the container, the difference in volume between the CPA solution and the container could affect the thermomechanical stresses formed during vitrification and subsequent rewarming.

### Human heart model

The human heart model is three orders of magnitude larger organ than the rat heart, Table 1, which introduces new challenges from the perspective of the thermal sciences, in addition to the obvious scale-up clinical challenges. Rewarming of the human heart is divided into three steps: (i) an initial slow convective rewarming below *T*_*g*_, to minimize thermomechanical stress in the vitrified system; (ii) temperature hold around glass transition to minimize stress at the onset of rewarming above *T*_*g*_ [34]; and (iii) rapid nanowarming combined with convective heating, as discussed above.

**Fig 8:**
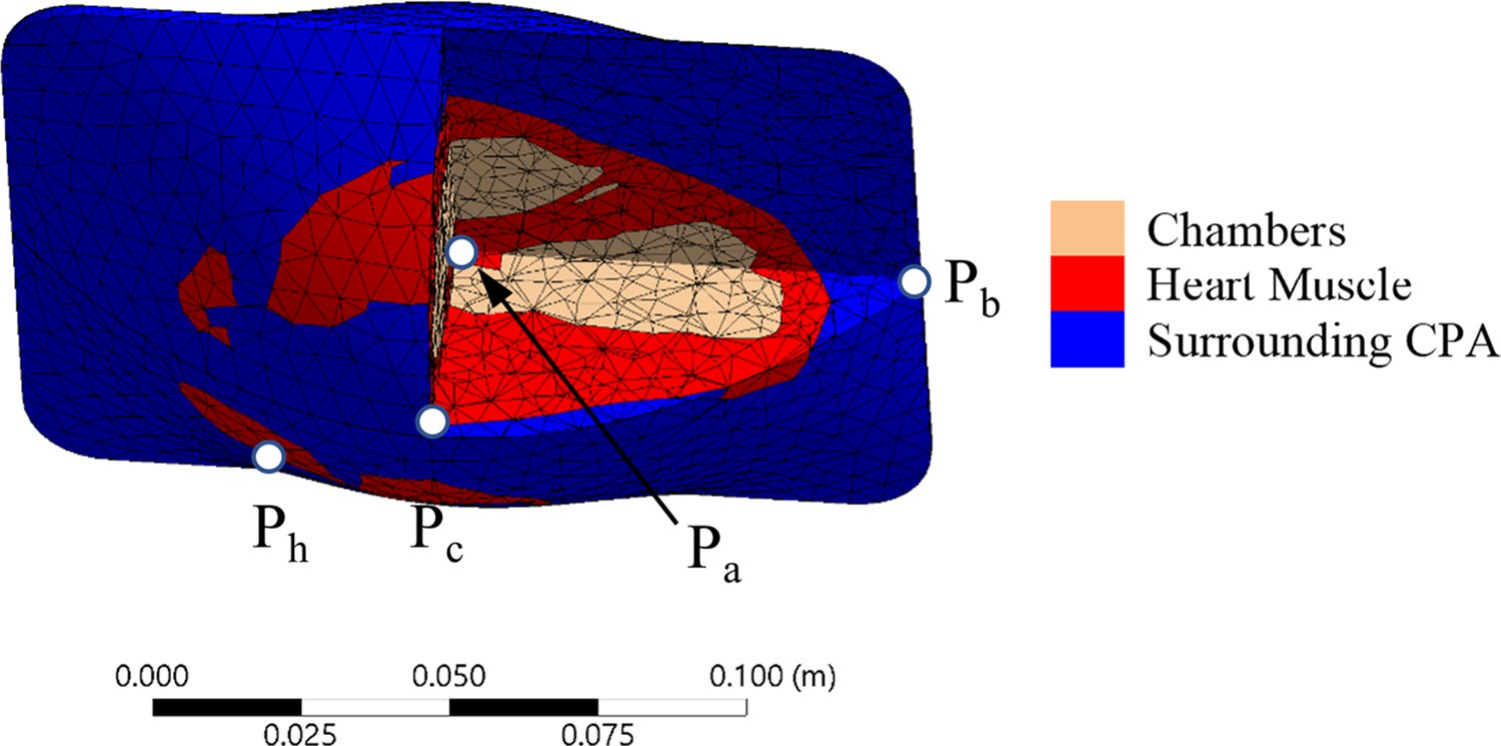
FEA mesh for the human heart model, along with the four representative points, where P_a_ is the volumetric center, P_b_ is the center of the short cryobag edge, P_c_ is the center of the bag’s surface, and P_h_ is the location where the maximum stress occurs during nanowarming.

Fig 8 displays the FEA mesh for a case study of the human heart, where the geometric locations of three reference points are similar to those in the rat heart, while a fourth point, P_h_, is selected at the location where *σ*_*MP*,*max*_ occurs during rewarming. The thermal protocol selected for this case is: (a) an initial cooling at a constant rate of 40°C/min from an initial temperature of -30°C down to -118°C (3°C below *T*_*g*_ of VS55+0.6M sucrose [52]); (b) a temperature hold at -118°C until the specimen reaches thermal equilibrium to facilitate stress relaxation; (c) further cooling at a rate of 1°C/min to the cryogenic storage temperature of -150°C; (d) an indefinite temperature hold at cryogenic storage; (e) slow rewarming from a storage temperature of -150°C up to -116°C (1°C below *T*_*g*_), subject to a convective boundary condition while the chamber rewarms at 1°C/min; (f) a temperature hold when the boundary temperature reaches -116°C to achieve thermal equilibrium; and (g) nanowarming combined with convective heating, while the surroundings temperature increases at a rate of 5°C/min.

**Fig 9:**
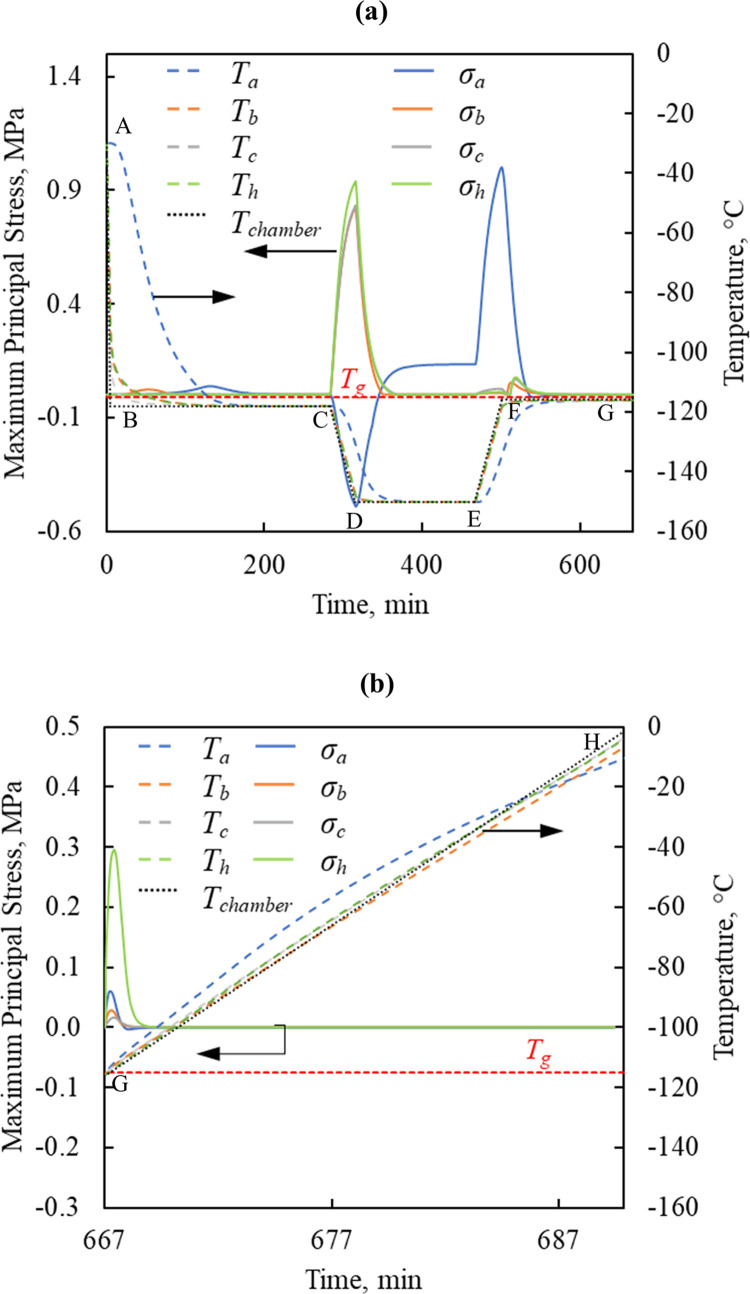
Thermal and stress history in the human heart model during (a) cooling and convective rewarming, and (b) nanowarming following the same cooling protocol in (a).

Fig 9 displays the thermal and stress histories at the four points of interest. Although the final cooling rate (Fig 9 segment C-D) in the chamber is set to 1°C/min for the human heart model and 40°C/min (Fig 3 segment C-D) for the rat heart model, the resulting *σ*_*MP*,*max*_ in the human heart (0.94MPa) is 30% higher than that in the rat heart (0.67MPa). Here, the size of the human heart (×270 the rat heart) [66–68] calls for a decreased cooling rate by a factor of 40. In broad terms, the magnitude of the stresses developed during cooling and rewarming are comparable, which is in fact the motivation behind the selected cooling and rewarming protocols.

With reference to Fig 9, *σ*_*MP*,*max*_ in tension occurs at the center of the specimen, P_a_, during the first step of rewarming. The stress decays during the following temperature hold step. During the third step of rewarming (nanowarming + convective heating), the maximum stress in tension occurs at P_h_. In fact, stresses developed during the nanowarming step were only 30% (0.3MPa) of those observed during cooling and convective rewarming, Fig 9. In the previous study, different thermal protocols were developed for the rat model and the human model, to ensure complete ice suppression in the very different organ sizes (27 folds difference). While the thermomechanical stresses were not known in that earlier study, those different thermal protocols also yielded comparable maximum stresses in the different organs sizes in the current study. For comparison, the stress developed in the human heart during nanowarming was 65% lower than that in the rat heart in Case III, which was the case that resulted in the lowest stresses in the rat heart model. It follows that size plays a dramatic role in the development of thermal protocols within the strength limits of the organ. While only one representative case of the human heart is modeled here, the size of the heart may vary significantly among people based on age, gender, body weight, physical shape, and across populations [69,70]. Furthermore, size matching is an important factor in heart transplantation [71]. With the demonstrated difficulties to design a thermal protocol to keep the thermomechanical stress within expected tolerable limits, it emphasizes the need to tailor the thermal protocol of the cryopreservation process to the heart on a case-by-case basis.

## Summary and conclusions

This study focuses on the analysis of thermomechanical stress in cryopreservation by vitrification of the heart, while exploring the effects of nanowarming-assisted recovery from cryogenic storage. This study follows a recent experimental investigation of cryopreservation on a rat heart model [35], and a theoretical thermal study of cryopreservation by vitrification on rat and human models [43]. Specifically, this study focuses on scenarios with variable sIONP concentration based on the physiology of the heart, while addressing heart scaling effects and container shape and size. The study focuses on the unique conditions associated with the rat heart model, contained in a cryobag as a choice of practice. Additionally, this study brings a simulated example of thermomechanical stresses in the human heart model, while addressing scale up concerns. To simplify the problem, this study addresses only the case of a highly complaint container as the best-case scenario, while acknowledging that stiffer container walls might elevate the maximum mechanical stress in the system and might shift the location of the peak stress in the domain.

Results of this study suggest that variable sIONP concentration based on the heart physiology will elevate mechanical stresses when compared with the mathematically simplified, uniform distribution case. The most dangerous part of rewarming is below glass transition and at the onset of nanowarming past the glass transition temperature on the way for organ recovery from cryogenic storage. At higher cryogenic temperatures, say 10°C above glass transition, the observed stresses are negligible, but this temperature interval is dependent on the rewarming rate, and hence the mechanical strain rate. It follows that the thermal gradient at the end of nanowarming is not an indication of stresses generated in the domain, but instead, the thermal gradient at the onset of nanowarming is the critical measure. One possibility to modulate the stress level at that stage is applying variable nanowarming power, but the required power modulation is a function of nanoparticle distribution, physiology of the heart, and geometric considerations.

Throughout rewarming, regions that rewarm faster, such as the chambers of the heart (higher sIONP concentration), undergo compressive stresses, while the slower rewarming regions, such as the heart myocardium (low sIONP concentration), undergo tension. Being a brittle material, the vitrified organ is expected to fail under tension in lower stresses than in compression [33,46]. The location and magnitude of the maximum stress in the investigated cases varied, while general rules were not identified. However, matching the convective rewarming effect on the surface of the container with the nanowarming effect and geometry of the problem may mitigate excessive stresses. This observation makes the thermal boundary condition an important design parameter for the cryopreservation protocol, not only from the thermal considerations but also from the mechanics considerations.

While this study begins with the rat heart model, being a follow up study on prior experimental [35] and theoretical investigations [43], a representative case of the human heart is further investigated to demonstrate the challenges associated with heart cryopreservation scale-up (two orders of magnitude larger). This investigation demonstrates the need to tailor the thermal protocol of heart cryopreservation on a case-by-case basis due to a host of factors, among which are: (i) size matching as an important factor in heart transplantation [71]; (ii) heart size variation among people based on age, gender, body weight, physical shape, and across populations [69,70]; (iii) geometry of the container; (iv) type, concentration, and loading capability of nanoparticles; and (v) physical properties of the CPA. Furthermore, the location and direction of the maximum stress in the process cannot be predicted *a priori*, nor the time that it reaches peak value. While thermomechanical stress poses a significant risk to organ integrity, careful design of the thermal protocol can be instrumental in reducing the likelihood of structural damage.
